# Analogue magnitude representation of angles and its relation to geometric expertise

**DOI:** 10.1038/s41598-024-59521-6

**Published:** 2024-04-18

**Authors:** Mateusz Hohol, Piotr Szymanek, Krzysztof Cipora

**Affiliations:** 1 Mathematical Cognition and Learning Lab, Copernicus Center for Interdisciplinary Studies, Jagiellonian University, Krakow, Poland; 2 Doctoral School in the Social Sciences, Jagiellonian University, Krakow, Poland; 3 Centre for Mathematical Cognition, Loughborough University, Loughborough, UK

**Keywords:** Psychology, Human behaviour

## Abstract

The distance effect (comparing objects becomes easier with increasing differences in their magnitude) is observed in tasks ranging across domains, and its existence has been interpreted as evidence for analogue magnitude representation. Similarly, associations between response side and magnitude (faster left/right-sided responses to small/large objects, respectively) are observed across domains. We investigated the analogue processing of angles and the association between angle magnitude and response side in relation to geometric expertise. We compared the behavioural pattern of two groups—architects and controls—in a direct angle magnitude classification task (i.e., judge whether a presented angle was greater or less than 90°) and in an indirect task (i.e., judge whether an angle was drawn with a dashed or continuous line). We found a robust distance effect for reaction times and accuracy at the whole sample level and in each group separately. Architects revealed a smaller distance effect for accuracy than controls. This could be interpreted as an argument for a more precise analogue representation of angles in experts compared to non-experts. However, we did not find evidence for an association between angle magnitude and response side in any group.

## Introduction

We encounter multiple symbolic and non-symbolic magnitudes in our daily lives. Many researchers believe that analogue representation, i.e., a single evolutionarily ancient and culturally universal mode of processing^1,2^ underlies all magnitude processing. A theory of magnitude (ATOM) postulates the existence of shared cortical metrics of multiple magnitudes^3,4^ and sheds light on well-documented similarities between space, numbers, and other magnitudes processing. Still, in practice, not all magnitudes are investigated equally often. Despite the longstanding research tradition^5–7^, the angle processing has been studied relatively rarely in contemporary experimental psychology with few notable exceptions^8–15^. This is surprising since the angle is one of the most fundamental geometric properties that need to be properly used and applied in science, engineering, and architecture^16^. From previous studies, we learn, for example, that the size of small angles is overestimated, and the size of big angles is underestimated^6,7^. Moreover, although angle discrimination generally obeys Weber’s law, a right angle is detected more precisely than other angles^15^, indicating that the category of perpendicularity has strong perceptual foundations^8^.

### Analogue magnitude processing

The postulate of the existence of the basic magnitude representation is supported by a large body of evidence for shared behavioural characteristics of comparative judgements. These characteristics include a well-established psychophysical phenomenon called “the distance effect,” occurring for reaction time (henceforth, RT) and accuracy. When comparing two objects, both humans and non-human animals take longer and make more mistakes when the objects to be compared are very similar in their magnitude than when the difference gets larger^1,2,17^. In humans, such a pattern has been found, among others, in comparisons/classifications of non-symbolic numerosities^18^, line lengths^19^, object sizes^20^ and luminance^21^. Recent studies also indicate that the internal structure of conceptual models of ordered objects is characterized by the (symbolic) distance effect^22^. Moyer and Landauer^23^ observed the distance effect in the case of numerical comparative judgments. Similarly, the distance effect can be observed when participants judge whether two numbers are equal or not (the same-different task)^24^ and classify whether a presented number is bigger or less than a fixed target number (magnitude classification task)^25^. The phenomenon is universal for positive and negative numbers^26^. Moreover, as recently shown, the distance effect could appear in grip force fluctuations even if the explicit motor response is not required in the magnitude classification task^27^.

According to the most common interpretation, as the participant’s performance in comparison/classification tasks follows Weber’s law, the distance effect reflects the analogue mode of the magnitude processing^28^. A smaller numerical distance effect is frequently interpreted as a marker of a more precise magnitude representation that could serve as a scaffolding for more advanced mathematical cognition, though the findings on that matter are mixed^25,29,30^. Furthermore, the distance effect on Arabic numbers has been traditionally explained in the context of “the Mental Number Line” (MNL), namely, the spatially organized representation of numbers, where smaller values are placed on the left and larger on the right^31,32^. According to this line of investigation, the smaller distance effect indicates a more precise representation of the MNL. However, as the magnitude representation can be activated independently from a spatial association, it is possible to account for the numerical distance effect as a non-spatial phenomenon^33,34^.

### Directional spatial mapping of magnitudes

Even though the distance effect could be considered an instance of analogue but not necessarily spatial processing, the theoretical construct of the MNL is supported by a directional association between the response side and numerical magnitude^31,35,36^. In speeded bimanual tasks where participants make judgments about the numbers, their reactions are faster to small numbers with the left hand and to large numbers with the right hand. This phenomenon has been dubbed the SNARC effect (Spatial-Numerical Association of Response Codes)^37^ and has been replicated numerous times using different methodologies^27,38,39^. The two most popular tasks that allow observing the SNARC effect include the magnitude classification (see previous section) and the parity judgment task (participants judge whether a presented number is even or odd). While mapping number magnitude into space is supposed to have deep evolutionary origins^40^, several cultural and education-related variables contribute to the strength of the original SNARC effect, including reading/writing direction, finger counting habits, and mathematical skills^38,39^. Despite the findings showing that the relationship between the SNARC effect and the latter variable is ambiguous^38^, students with a scientific background and professional mathematicians tend to reveal a weaker SNARC effect or no effect at all, respectively^37,41^.

Several studies investigated associations between the response side and magnitudes other than symbolic numbers^42^. SNARC-like compatibility effects have been found for non-symbolic numerosities^43,44^ and magnitudes, including physical^45,46^ and conceptual object size^47,48^, auditory pitch height^49,50^, musical note values^51,52^, luminance^53^, and—most importantly for the present study—angles^10^. The SNARC-like effect found in the latter study has a reversed direction compared to the classic SNARC effect, i.e., faster left/right-hand responses to large/small angles, respectively. Fumarola et al.^10^ pointed out that during school education, graphical representations of angles are introduced and drawn with a counterclockwise progression, i.e., right side orientation of the upper of two arms against the vertex in angles smaller than the right angle, and left side orientation of this arm in angles larger than the right angle. Importantly, Fumarola et al.^10^ found the effect in the group of civil engineering students (high familiarity with angles) but not in psychology students (low familiarity with angles) and interpreted this observation so that high familiarity with angles drives the association between the response side and angle magnitude. This pattern differs from findings on the classic SNARC (weaker SNARC associated with higher math skills in adults) but resembles the SNARC-like effects found in musicians regarding music notation^51^ and auditory pitch height^49,50^.

### The present study

Our aim was to (1) investigate the analogue processing of angle magnitudes, measured in terms of the distance effect, and (2) revisit the spatial-angle association indicated by the SNARC-like effect^10^. In both cases, we wanted to look at the effects of interest in relation to geometric expertise. To this end, we recruited two groups: professional architects (high familiarity with angles) and students of social sciences (low familiarity with angles). Noteworthy, we focused on architects as professionals with years of training in applied geometry and high exposition to angles in their daily work. By doing this, we applied the strategy of testing extreme groups within a given domain that has provided instructive insights into several fields of psychological science^54^. Also, the field of mathematical cognition has gained valuable insights by testing extreme groups^55–57^. Architects have been studied only in a few psychological studies^58,59^, and none of those studies focused on the basic processing of angles. Here, participants performed two computerized tasks modeled on those used in Fumarola et al.’s^10^ study. In the direct angle magnitude classification task, which allows measuring both the distance effect and the SNARC-like effect, participants assessed whether a presented angle was greater or less than 90°. In the indirect task used to measure the SNARC-like effect, they decided whether an angle was drawn with a dashed or continuous line.

Our hypotheses were the following: First, as the distance effect characterizes the analogue processing of many magnitudes, we expected to observe it in the direct task at the whole sample level and in each group separately. Second, we hypothesized that architects reveal a weaker distance effect on angle magnitudes than controls. We expected that costs related to more difficult vs. easy tasks could be operationalised in terms of response accuracy and speed. Having a more refined mechanism of dealing with a specific task (i.e., architects dealing more with angles) may thus be associated with a smaller increase in the cost when dealing with more challenging items (which, to a degree, is in line with observations that ratio-effect in non-symbolic comparisons is related to mathematical skills)^30^. Similarly, a smaller distance effect in the case of numerical stimuli is frequently interpreted as indicating a more precise magnitude representation^60^, but see^25^. Third, we expected to conceptually replicate the findings of Fumarola et al.^10^. Specifically, we predicted finding the right-to-left SNARC-like effect in the group of architects in both tasks. Considering that architects not only receive training in the field of applied geometry but also their daily professional activity involves extensive practice with angles, we hypothesized that the SNARC-like effect in this group would be more pronounced compared to the one found by Fumarola et al.^10^ on civil engineering students.

## Methods

### Participants

Two groups were recruited: professional architects and controls. The inclusion criteria for the first group were holding an MSc in architecture and working in an architectural design studio as an architect. The control group was recruited mainly from students of social sciences, all without experience in geometry going beyond the high school curriculum. Overall, 62 participants were recruited (37 women and 25 men; *M* = 30.9 years, *SD* = 9.96). Half of them were architects, and half were controls. Data from 5 individuals were excluded from the analysis of the direct task due to excessive error rates and timeouts (< 70% valid trials). Thus, we analysed data from 57 participants (33 females, 24 males) aged 18–62 years (*M* = 31 years, *SD* = 10.1): 31 architects (15 females, 16 males; *M* = 36 years; *SD* = 9.91) and 26 controls (18 females, 8 males; *M* = 25 years; *SD* = 6.52). Data from 2 participants were excluded from the indirect task, thus we analysed data from 60 participants (35 females, 25 males) aged 18–62 years (*M* = 31.1 years, *SD* = 10.1): 31 architects (15 females, 16 males; *M* = 36 years; *SD* = 9.91), 29 controls (20 females, 9 males; *M* = 25.7 years; *SD* = 7.26). All the participants had normal or corrected-to-normal vision and self-reported being right-handed native Polish speakers. All participants provided written informed consent and were informed that they could withdraw from participating in the study at any time without giving a reason. The study conformed to the Declaration of Helsinki and local guidelines for testing human participants.

### Materials

The participants completed two computerized behavioural tasks. In the direct angle magnitude classification task, they assessed whether a presented angle was greater or less than 90°. In the indirect task, where angle magnitude is not relevant, participants decided whether an angle was dashed or continuous. In both tasks, the following angles were presented: 30°, 45°, 60°, 75°, 105°, 120°, 135°, and 150° (the right angle was not presented in any trial). Participants responded by pressing Z or M buttons on a standard QWERTY keyboard. Both speed and accuracy were emphasized in the instruction. All angles were presented in black against a white background. In each task, half of the angles were presented with continuous and half with dashed line type. All angles were presented so that the bisector was vertical (but not visible to the participant). In half of the trials, the arms of the angle were facing upwards and in the other half, they were facing downwards. Each task contained two blocks with reverse response key mappings. In each block, each angle was presented 20 times to each participant (8 angles × 20 repetitions × 2 blocks × 2 tasks = 640 trials). The trial order was randomized with the restriction that each angle could not appear more than two times in a row. Short practice sessions preceded blocks (each consisting of 9 trials). In practice sessions, accuracy feedback was presented following incorrect responses, and information about response mapping was present in the bottom line of the screen. The order of tasks and blocks was counterbalanced among participants. In experimental blocks, no feedback and no information about the response key assignment was present. Each trial started with an eye fixation cross presented for 400 ms. Subsequently, an angle was presented until the participant responded or for a maximum duration of 1800 ms. The next trial started after 500 ms of blank screen presentation.

Noteworthy, the tasks were modelled on the ones used in Fumarola et al.’s study^10^. The main difference between Fumarola et al.’s task and our task was the number of experimental trials (8 angles × 10 repetitions × 2 blocks × 2 tasks = 320 trials in the former study). Moreover, contrary to Fumarola et al., we decided to provide no accuracy feedback in experimental trials.

Experimental tasks were implemented in the Inquisit v4^61^ and ran on Microsoft Windows-compatible portable computers with 15.4-inch screens. The tasks are shared at the Open Science Framework (https://osf.io/ycdr9/).

### Procedure

All the participants were tested in group settings: controls at the university in the lab, and architects in quiet rooms of design studios where they work. We did our best to make the testing conditions of both groups similar. At the beginning of each session, informed consent was obtained from each participant. Subsequently, participants sat in front of the computer (the distance from the screen was about 50 cm) and performed computerized tasks. The procedure lasted around 20 min.

### Analysis

#### Preprocessing and estimating the reliability (direct and indirect task)

Data processing and analyses were conducted in the R language (v. 4.3.1)^62^ using RStudio (v. 2023.06.0 Build 421)^63^. We used the following packages: jmv^64^, reshape2^65^, plyr^66^, dplyr^67^, psych^68^, tidyr^69^, ggplot2^70^, patchwork^71^. Both the data and analysis script are available at the Open Science Framework (https://osf.io/ycdr9/). Data from the practice sessions was not analysed. Data preprocessing was run in the same way for both tasks. First, we excluded participants whose error and timeout rates exceeded the 3 SD relative to the sample mean. Second, we excluded trials with incorrect responses. Third, we filtered RT data: RTs < 200 ms were treated as anticipations and not further analysed. Subsequently, we applied a sequential trimming method (the same as in Hohol et al.’s study^72^) to exclude outlier RTs: After calculating RTs and SDs for each participant separately, we removed RTs outside ± 3 SD from a participant mean. This procedure was repeated until no further changes in means and SDs existed. Ultimately, 87% of data from the direct task and 91% from the indirect task were considered in the main analysis. To check for the stability of our data, we estimated the reliability of the distance effect and the SNARC-like effect in the direct task and the SNARC-like effect in the indirect task. To this end, we used a split-half method (Spearman–Brown corrected; see the Suppl. Material to^73^).

#### Calculating the distance effect for RT and the distance effect for accuracy (direct task)

As the distance effect on angle magnitudes is considered to be analogous to the numerical distance effect, we applied a similar method to the one used frequently in numerical cognition studies^25^. For each angle we calculated the distance from 90° (i.e., 30° and 150° are in the distance of 60°; 45° and 135° of 45°; 60° and 120° of 30°; 75° and 105° of 15°). For each participant we calculated mean RT for each angle and regressed it on the distance and the size of the angle. For the distance effect we considered slopes associated with the distance predictor (note that the slopes associated with the size of the angle were used for calculating the size effect; see Supplementary Material 2). The negative slopes correspond to the typical distance effect, and more negative slopes indicate a stronger effect. To test for the distance effect at the whole sample level and in each group separately, we tested slopes against 0 by means of the one-sample *t*-test (one-sided, μ < 0). We investigated potential differences in distance slopes between architects and controls by means of independent samples *t*-test (two-sided). We performed the same analyses for accuracy (the proportion of correct trials was included in the regression instead of the mean RT, and the hypothesized sign of the slopes was reversed). In addition to frequentist analyses, we also performed Bayesian *t*-tests in jmv package^64^, which allowed us to quantify evidence for existing (BF_10_) and null effects (BF_01_ = 1/BF_10_). In all the Bayesian analyses we used default Cauchy prior distribution with a scaling of 0.707. We interpreted BF values using categories proposed by Jeffreys^74^ with labels updated by Wetzels et al.^75^.

The task also allows for investigation of the size effect (less efficient processing of large objects than small objects)^9,76^ and the ratio effect (performance decreases with increasing magnitude ratio)^77,78^, which also characterize analogue magnitude processing. A full report of these phenomena and descriptions of methods of calculating them are provided in Supplementary Materials 1 and 2.

#### Calculating the SNARC-like effect (direct and indirect tasks)

The effect of interest is considered to be the angle magnitude-related equivalent of the classic SNARC, with the caveat that the angle magnitude progression is reversed, i.e., from right to left^10^. In line with Fumarola and collaborators, we applied a method of computing the SNARC effect proposed by Fias and colleagues^79^. This method considers unstandardized regression slopes as a measure of the effect and is frequently employed in numerical cognition studies^41,72^. First, we calculated the difference right-left hand in reaction time (dRT) for each angle magnitude for each participant. Positive dRTs indicate the left-hand advantage, and more negative ones show the right-hand advantage. Next, we regressed dRTs on angle magnitude. We considered regression slopes as a measure of the SNARC-like effect. To test for the SNARC-like effect at the whole sample level and in each group separately, we tested slopes against 0 with one-sample *t*-tests. Due to a direct prediction of the directionality of the SNARC-like effect^10^, we applied one-sided *t*-tests (μ > 0). To investigate potential differences in SNARC-like slopes between architects and controls, we performed an independent samples *t*-test. We also run Bayesian *t*-tests. We conducted the same analyses on data from the direct and indirect tasks. Note also that despite the lack of an explicit hypothesis, we calculated the effect for upward and downward-oriented angles separately (see Supplementary Material 4).

### Ethical approval waiver statement

According to “The recommendations of the Council of the National Science Centre, Poland on research involving human participants” (https://www.ncn.gov.pl/sites/default/files/pliki/2016_zalecenia_Rady_NCN_dot_etyki_badan.pdf), the approval of a local ethics committee is required in research in which: (1) participants have limited capacity to give informed consent to participate in the study (e.g., children before the age of twelve or participants are intentionally deceived); (2) participants are particularly vulnerable to trauma or mental health disorders; (3) active interventions to human behaviour (e.g., psychotherapy) are planned; (4) controversial issues or issues requiring particular sensitivity and consideration to be investigated; and (5) long-lasting, tiring, physically or mentally fatiguing tests are planned. The study presented here does not meet any of these conditions, and thus, the approval of a local ethics committee is not required. In our study all the participants were healthy and legally aged and given their informed consent. No masking instruction or any other form of deception was applied. Participants did not belong to any of the groups particularly vulnerable to trauma or mental health disorders. The study did not address any of the controversial issues and did not involve active behavioural interventions: the procedure consisted of observing angles presented on the screen and reacting to them by pressing keyboard keys and lasted around 20 min. The study conformed to the Declaration of Helsinki.

## Results

### The direct task

#### Overview and reliability

The summary is presented in the left part of Table 1. The overall accuracy in the direct task was 93% and differed significantly between groups in the frequentist *t*-test (*t*_55_ = 2.16, *p* = 0.035, Cohen’s *d* = 0.57), with anecdotal Bayesian support (BF_10_ = 1.80) for architects being more accurate than controls. Since we found (expected) ceiling effects in the accuracy, we applied [2*arcsin(sqrt(proportion_correct))] transformation to check for the robustness of our conclusions and to mitigate the problem of the non-normality of the data distribution. The analyses on the transformed accuracy data also revealed significant differences between groups (*t*_55_ = 2.39, *p* = 0.020, *d* = 0.64) with anecdotal support (BF_10_ = 2.75) for architects being more accurate than controls. The overall RT was 537.51 ms (SD = 70.71) and did not differ significantly between groups (*t*_55_ = 1.60, *p* = 0.116, *d* = 0.42; BF_01_ = 1.30). The intraindividual variability in RT was 140.37 ms (SD = 43.63) and did not differ significantly between groups (*t*_55_ = 0.92, *p* = 0.359, *d* = 0.25; BF_01_ = 2.60). Split-half, Spearman–Brown corrected reliabilities were 0.93 for the distance effect and 0.92 for the SNARC-like effect.

**Table 1:** Characteristics of accuracies and reaction times.

Group	Direct task				Indirect task			
	Accuracy (%)	Trans. ACC	RT (SD)	I-V (SD)	Accuracy (%)	Trans. ACC	RT (SD)	I-V (SD)
Whole sample	93	2.64	537.51 (70.71)	140.37 (43.63)	97	2.83	439.71 (63.97)	86.86 (27.35)
Architects	94	2.70	551.03 (82.22)	145.27 (51.80)	97	2.85	459.73 (69.19)	91.71 (30.82)
Controls	91	2.57	521.40 (50.88)	134.53 (31.28)	96	2.80	418.30 (50.74)	81.67 (22.46)

Trans. ACC refers to the [2*arcsin(sqrt(proportion correct))] transformed accuracy; I-V refers to the intraindividual variability in RT.

#### The distance effect for RT

**Figure 1:**
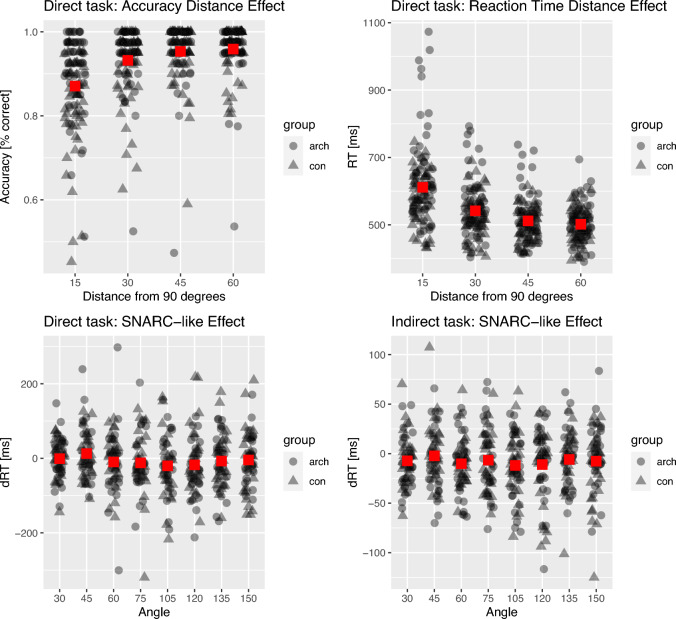
Summary of main findings. Top part: Distance effect in the direct task. Accuracy-based (left panel) and reaction time-based (right panel). X-axes represent the distance from the reference angle of 90 degrees. Y-axes represent percentages of correct reactions and reaction times, respectively. Bottom part: SNARC-like effects in the direct (left panel) and the indirect (right panel) tasks. X-axes represent angle magnitude, and Y-axes represent dRT (RT right hand − RT left hand). In each panel, group means for a specific distance/angle magnitude are presented with red squares, while individual data points are presented with dots (architects) and triangles (control group).

Since we did not find a significant correlation between overall RT and accuracy (*r* = − 0.07, *p* = 0.616), we calculated the distance effect using both performance measures separately^80^. The results are summarized in Table 2 and in Fig. 1. The analyses revealed a robust distance effect for RT at the whole sample level and in each group separately, with marginally significant between-group differences (*t*_55_ = − 2.02, *p* = 0.048, *d* = − 0.54). The Bayesian equivalent of the independent samples *t*-test showed anecdotal support for the alternative hypothesis (BF_10_ = 1.43). Even though when tested against zero, the effect size associated with the distance effect was larger in controls than in architects, the mean distance effect slope was larger for controls, and the direct comparison revealed the effect to be stronger in architects. These were due to two outliers in the architect groups demonstrating very strong distance effects (which, in consequence, increased variance in this group, decreasing the effect size when testing against zero). When these outliers were not considered in the analysis, the effect size for architects got larger, however, the between-group difference was no longer present (see Supplementary Material 3).

**Table 2:** The distance effect for RT, the distance effect for accuracy, and SNARC-like effect slopes. Significant values are in bold.

Group	Distance slopes for RT (direct task)				Distance slopes for ACC (direct task)				SNARC-like slopes (direct task)				SNARC-like slopes (indirect task)			
	Mean (SD)	*t*-test	*d*	BF_10_	Mean (SD)	*t*-test	*d*	BF_10_	Mean (SD)	*t*-test	*d*	BF_01_	Mean (SD)	*t*-test	*d*	BF_01_
Whole sample	− 2.40 (1.75)	***t***_**56**_** = **− **10.33, *p* < 0.001**	− 1.37	> 10^11^	0.0019 (0.0015)	***t***_**56**_** = 9.55 (*p* < 0.001)**	1.26	> 10^10^	− 0.11 (1.22)	*t*_56_ = − 0.68, *p* = 0.749	− 0.09	10.88	− 0.02 (0.29)	*t*_59_ = − 0.58, *p* = 0.719	− 0.08	10.55
Architects	− 2.81 (2.10)	***t***_**30**_** = **− **7.44, *p* < 0.001**	− 1.34	> 10^6^	0.0011 (0.0009)	***t***_**30**_** = 7.08 (*p* < 0.001)**	1.27	> 10^5^	− 0.11 (1.19)	*t*_30_ = − 0.50, *p* = 0.688	− 0.09	7.32	0.03 (0.26)	*t*_30_ = 0.59, *p* = 0.280	0.11	3.12
Controls	− 1.90 (1.04)	***t***_**25**_** = **− **9.31, *p* < 0.001**	− 1.83	> 10^7^	0.0027 (0.0016)	***t***_**25**_** = 8.73, *p* < 0.001**	1.71	> 10^6^	− 0.11 (1.29)	*t*_25_ = − 0.45, *p* = 0.673	− 0.09	6.58	− 0.07 (0.31)	*t*_28_ = − 1.28, *p* = 0.895	− 0.24	10.59

*t*-test = one-sample *t*-test against zero (one-sided); significant results are marked with a bold font. *d* = Cohen’s *d*; BF = Bayes factor; for the SNARC-like effect BF_01_ values are provided; for the distance effects BF_10_ values are provided (BF_01_ = 1/BF_10_).

Our groups differed in age. This might have affected between-group differences in the effects of interest. To ensure this was not the case, we calculated the same effects using z-transformed RTs, as comparing these would be more robust to differences in overall RTs. The results of the analysis of the distance effect on *z*-scored RTs did not differ from those obtained using unstandardized RTs (see Supplementary Material 4). However, again, the difference relied only on an outlier in the architects group.

#### The distance effect for accuracy

The results are summarized in Table 2 and in Fig. 1. The distance effect for accuracy was significant at the whole sample level and in each group taken separately. Groups differed significantly in the frequentist analysis (*t*_55_ = − 4.65, *p* < 0.001, *d* = − 1.24) along with decisive Bayesian evidence (BF_10_ = 855.08). To check the robustness of these results, we calculated the distance effect for accuracy on [2*arcsin(sqrt(proportion_correct))] transformed data and found a similar between-group difference (*t*_55_ = − 4.70, *p* < 0.001, *d* = − 1.25; BF = 1012.17).

#### The SNARC-like effect

The results are summarized in Table 2 and in Fig. 1. Frequentist analyses did not reveal a significant SNARC-like effect at the whole sample level nor in each group separately. Bayesian analyses delivered substantial evidence for the null hypothesis in each case. We did not find between-group differences in SNARC-like slopes (*t*_55_ = 0.03, *p* = 0.979, *d* < 0.01) in the frequentist analysis, while the Bayesian analysis revealed substantial evidence for the null (BF_01_ = 3.72). In Supplementary Material 4, we also report the results of analyses conducted separately for upside and downward-oriented angles. They did not show the SNARC-like effect in any orientation in any group.

### The indirect task

#### Overview and reliability

The summary is presented in the right part of Table 1. The overall accuracy in the indirect task was 97% and did not differ significantly between the groups (*t*_58_ = 0.62, *p* = 0.540, *d* = 0.16; BF_01_ = 3.25). We also did not find between-group differences when the transformed accuracy data [2*arcsin(sqrt(proportion_correct))] were taken into account (*t*_58_ = 1.50, *p* = 0.139, *d* = 0.39; BF_01_ = 1.49). The overall RT was 439.71 ms (SD = 63.97) and differed significantly between groups (*t*_58_ = 2.63, *p* = 0.011, *d* = 0.68; BF_10_ = 4.39). The intraindividual variability in RT was 86.86 ms (SD = 27.35) and did not differ significantly between groups (*t*_58_ = 1.43, *p* = 0.157, *d* = 0.37; BF_01_ = 1.62). We did not find a significant correlation between overall RT and accuracy (*r* = 0.12, *p* = 0.354). The split-half, Spearman–Brown corrected reliability of the SNARC-like effect was 0.16.

#### The SNARC-like effect

The results are summarized in Table 2 and in Fig. 1. Frequentist analyses did not reveal a significant SNARC-like effect at the whole sample level nor in each group separately. Bayesian analyses delivered substantial evidence for the null hypothesis in each case. In frequentist analysis, we did not find between-group differences in SNARC-like slopes (*t*_58_ = 1.38, *p* = 0.174, *d* = 0.36). The Bayesian *t-*test showed anecdotal evidence for the null (BF_01_ = 1.73). In Supplementary Material 4, we also report the results of analyses conducted separately for upside and downward-oriented angles. They did not show the SNARC-like effect in any orientation.

## Discussion

### Overview

We focused on two aspects of the cognitive processing of angles: analogue magnitude representation and the association between the response sides and magnitude, along with their relationship to expertise in applied geometry. We tested two groups: professional architects and social sciences students, who performed two tasks. The direct task required classifying whether an angle was greater or less than the right angle, and the indirect task required judging whether an angle was presented as dashed or continuous line. We expected to find the distance effect in both groups, wherein we hypothesized that architects reveal a weaker effect than controls. Moreover, we expected to replicate Fumarola et al.’s^10^ result, i.e., to find the right-to-left SNARC-like effect in the group of professionals characterized by high familiarity with angles and using applied geometry during daily work.

Since we did not find a significant correlation between overall RT and accuracy, we followed the guidelines presented by Bruyer and Brysbaert^80^ and calculated the distance effect for reaction times and accuracy separately. The distance effect for RT was present at the whole sample level and in each group separately in the direct task. In each case, the size of the effect was large. The pattern of differences in the distance effect for RT was complex (i.e., architects revealed numerically larger distance slope associated with smaller Cohen’s d than controls). The marginally significant effect was driven by outliers in the architects (who also attenuated Cohen’s d in this group when tested against zero; see Supplementary Material 3). At the same time, Bayesian analysis was largely inconclusive. Taking all these complexities into account, we do not make conclusions based on differences in the distance effect for RT between groups. At the same time, the results were more straightforward and conclusive in terms of the distance effect for accuracy. We find a large distance effect for accuracy at the whole sample level and in each group separately (which holds against all robustness checks—considering data transformation and outlier analysis). In both frequentist and Bayesian analysis, architects revealed weaker distance effect for accuracy than controls. Last but not least, in frequentist analyses, we did not find evidence for a SNARC-like effect in any group in any task. At the same time, we found substantial Bayesian evidence for the null. Thus, we could not replicate Fumarola et al.’s^10^ results.

### The analogue magnitude representation of angles: the distance effect

Basic physical magnitudes humans and other animals encounter in the surrounding world are encoded in the analogue format, and their discrimination follows Weber’s law^28^. It has been proposed that in human beings, all the magnitude processing, including the processing of symbolic numbers, originates from the single analogue magnitude system implemented in the parietal cortex^3,4^. This claim is substantiated in neuroimaging findings that the intraparietal sulcus is sensitive to angles, lines, and symbolic numbers^9^ and in shared psychophysical characteristics of magnitude comparisons/classifications. These characteristics include the distance effect occurring for stimuli such as symbolic numbers^23^, non-symbolic numerosities^18^, object sizes^20^, line lengths^19^, or luminance^21^. Although it has been demonstrated that angle discrimination obeys Weber’s law^15^, to our knowledge, the distance effect on angle magnitudes has never been explicitly reported in the literature. On the other hand, not all researchers agree that the same mechanism drives all just mentioned occurrences of the distance effect. For instance, according to Krajcsi et al.^81^, while in comparisons/classifications of non-symbolic magnitudes (including non-symbolic numerosities), the distance effect emerges from a shared analogue representational mechanism, the corresponding phenomenon for symbolic numbers reflects the semantic distance between the numbers stored as nodes in so-called Discrete Semantic System being a kind of the mental lexicon.

In mathematical cognition literature, it has been proposed that a smaller distance effect (and a smaller ratio effect; see Supplementary Material 1) corresponds with a more precise representation of a number that could serve as a building block of higher math skills^37,60,77,82,83^.

In line with this interpretation, in experts, a more refined mechanism of dealing with magnitudes may be associated with a smaller cost increase when dealing with more challenging items. According to an alternative, automatization-focused interpretation^84^, it is possible that non-experts could be more prone to a distance effect when solving magnitude classification tasks since they have to perform controlled memory searches/operations whose precision is correlated with the distance between to-be-classified magnitudes. On the other hand, a smaller distance effect in experts could reflect their ability (acquired in extensive practice) to retrieve a correct response from memory more automatically. Note, however, that professional mathematicians do not differ from non-mathematicians in the distance effect for symbolic Arabic numbers^25^. The ambiguity between this result and a smaller distance effect for angle magnitudes in architects reported here could be elucidated by separate scaffoldings for expertise in differing fields of math-related activity (symbolic numbers vs. geometry). Also, evidence for the negative association between the distance effect for non-symbolic numerosities and mathematical skills (lower effect, higher math, and vice versa) is somehow mixed^83,85,86^. Thus, our findings add the missing piece of evidence on the distance effect and suggest that expertise in applied geometry is associated with a more precise analogue representation of the angle or more automatic classifications of angles. However, this association and its interpretation should be investigated in the future.

### Attempt at replication of the SNARC-like effect

The SNARC effect is a robust phenomenon replicated many times^38,39^. Several studies revealed similar associations between the response sides and magnitudes^42^, including non-symbolic numerosities^43,44^, physical^45,46^ and conceptual object size^47,48^, auditory pitch height^49,50^, musical note values^51,52^, and luminance^53^. Thus, one might have expected such an association to occur in the case of the response side and angle magnitude. Indeed, Fumarola et al.^10^ found a right-to-left SNARC-like effect in civil engineering students and elucidated that the high familiarity with angles drives the phenomenon. Vis-à-vis previous findings showing that the relationship between a classic SNARC effect and mathematical competence is more evident in professional mathematicians^41^ than in students with a scientific background^37^, we expected to find even a stronger SNARC-like effect in professionals with years of training in applied geometry and high exposition to angles in daily practice.

We did not pose an explicit hypothesis regarding the differences in the probability of observing the SNARC-like effect in particular tasks. A classic SNARC effect occurs robustly in the magnitude classification task, where numerical magnitude is assessed directly, and in tasks where numerical magnitude is irrelevant but semantic processing of numbers is necessary to perform the task (e.g., the parity judgement task). It also appears, sometimes with a smaller effect size, in symbolic tasks when non-semantic features such as font type (upright vs. Italics) or colour are used as a response criterion^87–89^. However, identifying stimuli as numbers might be necessary for the colour judgment task to elicit the SNARC^90 ^(but see ^89^ for opposing results). Similarly, in th﻿e case of non-numerical magnitudes, SNARC-like phenomena occurring in implicit tasks tend to be smaller than those observed in direct tasks^42^. These tasks also do not require semantic processing of the stimuli (i.e., they are closer to font type/colour judgment than to parity judgment in the case of symbolic tasks). Moreover, in some studies, SNARC-like effects for time durations^91^ and weight^92^ have been observed in direct tasks but not indirect tasks. Even in the case of non-symbolic numerosities, SNARC-like effects tend to occur rather in direct^43^, than indirect tasks^93^. In the indirect task used by Fumarola et al.^10^ and in the present study, the lines do not need to be recognized as constituting the angle to be classified as continuous or dashed. Thus, one would expect the presence of the SNARC-like effect foremost in the task where the participants are directly asked to classify angle magnitudes. Nevertheless, although we used tasks similar to those of Fumarola et al. and doubled both the recruited sample and the number of trials per participant, we did not observe the SNARC-like effect in any group in any task.

Despite the lack of an explicit hypothesis, encouraged by one of the reviewers, we calculated the SNARC-like effect for upward and downward-oriented angles separately for exploratory purposes. Again, we did not find the effect of interest for any angle orientation in any group in any task (see Supplementary Material 5). According to the valuable suggestion of another reviewer, the SNARC-like effect observed by Fumarola et al.^10^ could be a short-living phenomenon that occurs in a given number of trials of the task and disappears later. To check this possibility, we repeated analyses of SNARC-like effects for the first half of trials in each task, which corresponds to the total number of trials in Fumarola et al.’s study. We did not find a SNARC-like effect in any of the tasks, neither at the whole sample level nor in any group. There were also no between-group differences in any task (see Supplementary Material 6). To sum up, our results do not support the claim that angles are represented along the MNL-like continuum, at least in terms of the association between angle magnitude and response side.

### Limitations of the study and future research directions

Finding a tendency toward weaker distance effects in architects than in controls could be interpreted in line with the claim that the former group reveals a more precise analogue magnitude representation. However, this supposition should be treated with caution since even in numerical cognition literature, as just mentioned, evidence for the association between the distance effect and mathematical skills is mixed. Next, while the responses of architects were generally slower but simultaneously more accurate in comparison to controls, we acknowledge that our groups differed in age. Thus, we also calculated the distance effect using *z*-scored reaction times and obtained similar results (at the sample level, in particular groups, and in terms of the lack of between-group difference) to those calculated using unstandardized RTs (see Supplementary Material 3). Next, only one of the tasks we used in our study allowed us to investigate the distance effect. Further studies should check the robustness of the association of the strength of the distance effect for angles with geometric expertise in other tasks, such as the angle magnitude comparison task, where two different angles will be presented simultaneously in a single trial. Finally, our design does not allow us to draw causal conclusions, i.e., it is not clear if a weaker distance effect emerges from the professional activity of architects or vice versa.

Regarding the SNARC-like effect, because of the changes we made in a number of trials and an experimental group, our attempt to reproduce Fumarola et al.’s^10^ results should be considered conceptual rather than direct replication^94^. Considering the difference in a classic SNARC effect between mathematicians and engineers reported previously^41^, the future direct replication of Fumarola et al.’s^10^ study would be desirable. What is more, our results remain silent regarding the existence of other kinds of associations between space and angle magnitude. Noteworthy, we learn from the numerical cognition literature that spatial-numerical associations constitute a broad range of phenomena, and a classic SNARC effect is just one of them^35,95^. Some researchers also claim that a classic SNARC effect does not reflect a persistent representation of numbers in spatial format but rather a temporary structure activated in working memory by the task^36^. Thus, future studies on the possible spatial representation of angles would develop novel tasks or adopt those used to investigate other phenomena classified as spatial-numerical associations. Last but not least, we acknowledge that while the reliabilities of the distance effect and the SNARC-like effect in the direct magnitude classification task were good in our study, the reliability of the SNARC-like effect in the indirect task was poor.

### Conclusions

We found evidence for the distance effect on angles, which enlarges the knowledge base on shared characteristics of magnitude processing. Moreover, we found weaker distance effects in architects than controls, which could indicate a more precise basic analogue magnitude representation in individuals with strong expertise in applied geometry. On the other hand, our results do not support the claim on the association between the angle magnitude and response side. The study adds to the rather scarce knowledge base on the processing of angle magnitudes in general and the even scarcer literature on the cognitive processes associated with professionals’ expertise in geometry.

### Supplementary Information

Supplementary Information.

## Data Availability

The data, analysis scripts, and experimental procedure are available at the Open Science Framework (https://osf.io/ycdr9/).
