# Supplementary material for: Analogue magnitude representation of angles and its relation to geometric expertise
Source: Sci Rep. 2024 Apr 18;14:8997. doi: 10.1038/s41598-024-59521-6 (PMC11026470; doi:10.1038/s41598-024-59521-6)
Supplement: Supplementary file 1 — Supplementary Information. [file 41598_2024_59521_MOESM1_ESM.docx]

Supplementary Materials to

**Analogue magnitude representation of angles and its relation to geometric expertise**

Mateusz Hohol, Piotr Szymanek, Krzysztof Cipora

**1. The ratio effect**

The ratio effect ^1^ is highly correlated with the distance effect, so many studies on the magnitude processing consider these effects interchangeably ^2^. To check the generality of our findings regarding the analogue magnitude processing of angles presented in the main paper, we repeated the analyses taking into account the ratio effect for RT and accuracy.

We used individual regression slopes to calculate the ratio effect, regressing mean RTs on the angle ratio. The ratio was calculated for each angle separately. It was the ratio of the presented angle and the right angle. To obtain the ratios for each angle and the criterion right angle, we divided the smaller angle by the larger one. This means angles 30°, 45°, 60°, 75° were divided by 90°, and 90° was divided by angles 105°, 120°, 135°, 150°. The ratios were 0.33, 0.5, 0.67, 0.83, 0.86, 0.75, 0.67, and 0.6, respectively, for angles 30°, 45°, 60°, 75°, 105°, 120°, 135°, and 150°. A more positive slope is interpreted as a stronger ratio effect. To test for the ratio effect at the group level, we tested slopes against 0 by means of the one-sample *t*-test (one-sided, μ > 0) and compared groups by means of independent samples *t*-test. We also used Bayesian equivalents of *t­*-tests.

The results of the slope analyses are summarized in Table S1. We found a robust ratio effect at the whole sample level and in each group taken separately, with the between-group difference not reaching statistical significance (*t*_55_ = 1.80, *p* = .078, Cohen’s *d* = 0.48). The Bayesian equivalent of the *t*-test remained inconclusive (BF_10_ = 1.01). Please note that similarly to the distance effect, there were outliers in the architects’ group, therefore, despite the numerically larger mean slope in this group, the effect size associated with a one-sample *t*-test is smaller in the case of this group. The ratio effect’s reliability (Split-half, Spearman-Brown corrected) was 0.92. Expectedly, the distance effect correlated with the ratio effect at *r* = -.97 (*p* < .001).

We also calculated the ratio effect for accuracy. It was significant at the whole sample level and in each group taken separately. Groups differed significantly in the frequentist analysis (*t*_55_ = 3.73, *p* < .001, *d* = 0.99), and the Bayesian evidence was decisive (BF_10_ = 59.76).

*Table S1*. The ratio effect, the ratio effect for accuracy, and the size effect slopes.

| Group | Ratio slopes for RT | | | | Ratio slopes for accuracy | | | | Size slopes for RT | | | |
| --- | --- | --- | --- | --- | --- | --- | --- | --- | --- | --- | --- | --- |
|  | Mean (SD) | *t*-test | *d* | BF_10_ | Mean (SD) | *t*-test | *d* | BF_10_ | Mean (SD) | *t*-test | *d* | BF_01_ |
| whole sample | 221.36  (164.14) | ***t*_56_ = 10.10,**  ***p* < .001** | 1.35 | >10^11^ | -0.15  (0.16) | ***t*_56_ = -7.13, *p* < .001** | -0.94 | >10^7^ | 0.002  (0.35) | *t*_56_ = 0.04,  *p* = .485 | 0.004 | 6.71 |
| architects | 256.41 (192.28) | ***t*_30_ = 7.42,**  ***p* < .001** | 1.35 | >10^5^ | -0.08 (0.16) | ***t*_30_= -3.05, *p* = .002** | -0.55 | 16.97 | -0.02 (0.29) | *t*_30_ = -0.33,  *p* = .628 | -0.06 | 6.59 |
| controls | 179.56 (112.42) | ***t*_25_= 8.14,**  ***p* < 0.001** | 1.60 | >10^6^ | -0.23 (0.13) | ***t*_25_ = -9.11,**  ***p* < 0.001** | -1.79 | >10^7^ | -0.02 (0.42) | *t*_25_ = 0.30  *p* = .383 | 0.06 | 3.79 |

*Notes.* *t*-test = one-sample *t*-test against zero (one-sided); significant results are marked with a bold font; *d* = Cohen’s *d*; BF = Bayes factor; for the ratio effects BF_10_ values are provided; for the size effect BF_01_ values are provided (BF_01_ = 1/BF_10_).

*Table S2*. SNARC-like slopes depending on the angle orientation (upward versus downward).

| Group | Upward (direct task) | | | | Downward (direct task) | | | | Upward (indirect task) | | | | Downward (indirect task) | | | |
| --- | --- | --- | --- | --- | --- | --- | --- | --- | --- | --- | --- | --- | --- | --- | --- | --- |
|  | Mean (SD) | *t*-test | *d* | BF_01_ | Mean (SD) | *t*-test | *d* | BF_01_ | Mean (SD) | *t*-test | *d* | BF_01_ | Mean (SD) | *t*-test | *d* | BF_01_ |
| Whole sample | -0.06  (1.37) | *t*_56_ = -0.34,  *p* = .663 | -0.05 | 8.84 | -0.12  (1.19) | *t*_56_ = -0.79, *p* = .782 | -0.10 | 11.56 | -0.02 (0.38) | *t*_59_ = -0.50,  *p* = .688 | -0.06 | 10.00 | -0.02 (0.33) | *t*_59_ = -0.43,  *p* = .665 | -0.06 | 9.58 |
| Architects | -0.08 (1.25) | *t*_30_ = -0.35,  *p* = .636 | -0.06 | 6.69 | -0.09 (1.27) | *t*_30_ = -0.40, *p* = .655 | -0.07 | 6.91 | 0.06 (0.34) | *t*_30_ = 0.91,  *p* = .185 | 0.16 | 2.22 | 0.01 (0.30) | *t*_30_ = 0.22,  *p* = .413 | 0.04 | 4.37 |
| Controls | -0.04 (1.53) | *t*_25_ = -0.14,  *p* = .555 | -0.03 | 5.35 | -0.16 (1.12) | *t*_25_ = -0.74,  *p* = .768 | -0.15 | 7.77 | -0.11 (0.41) | *t*_28_ = -1.44,  *p* = .919 | -0.27 | 11.29 | -0.05 (0.35) | *t*_28_ = -0.76,  *p* = .774 | -0.14 | 8.26 |

Notes. *t*-test = one-sample *t*-test against zero (one-sided); *d* = Cohen’s *d*; BF = Bayes factor. BF_01_ values are provided (BF_01_ = 1/BF_10_).

**2. The size effect**

The magnitude classification task also allows for calculating the size effect ^3^. Again, we used slopes of the angle size from the individual multiple regressions reported in the main text. Positive slopes correspond to the regular size effect ^4^. To test for the size effect at the group level, we tested slopes against 0 by means of the one-sample *t*-test (one-sided, μ > 0) and compared groups by means of independent samples *t*-test. We also used Bayesian equivalents of *t­*-tests.

The results are summarized in Table S1. Frequentist analyses did not reveal a significant size effect at the whole sample level nor in each group separately. Bayesian analyses revealed substantial evidence for the null hypothesis in each case. In frequentist analysis, we did not find between-group differences in size slopes (*t*_55_ = -0.44, *p* = .660, *d* = -0.18). The Bayesian *t-*test showed substantial evidence for the null (BF_01_ = 3.43). The reliability (Split-half, Spearman-Brown corrected) of the size effect was 0.81. To our knowledge, the size effect for angles was previously reported only by Fias et al. ^5^, and we did not reproduce this result.

**3. The distance effect without outliers in architects**

*The distance effect for RT*

In the left panel of Fig. S1, we can see that there are two outliers in the architects’ group. The distance effect for RT in architects holds and is more pronounced when removing these outliers (mean slope = -2.37, SD = 1.18 *t*_28_ = -10.77, *p* < .001, *d* = -2.00; BF_10_ > 10^9^), but the between-group difference disappears (*t*_53_ = -1.55, *p* = .126, *d* = -0.42; BF_01_ = 1.36).

*The distance effect for accuracy*

In the right panel of Fig. S1, we can see that there is one outlier in the control group. The distance effect for accuracy in controls holds and is stronger when removing the outlier (mean slope = 0.003, SD = 0.001; *t*_24_ = 10.26, *p* = < .001, *d* = 2.05; BF_10_ > 10^7^). The same holds regarding the between-group difference (*t*_54_ = -4.76, *p* < .001, *d* = -1.28; BF_10_ > 10^3^).

Figure S1. The distance effect for reaction times (left) and accuracy (right).

**4. The** **distance effect on** ***z*-scored reaction times**

The reliability (Split-half, Spearman-Brown corrected) of the distance effect calculated on *z*-scored RTs was 0.80. The mean values of distance slopes were -0.016 (SD = 0.007) for the whole sample, -0.018 (SD = 0.007) for architects, and -0.014 (SD = 0.007) for controls. We found a robust distance effect for *z*-scored RTs at the whole sample level (*t*_56_ = -17.21, *p* < .001, *d* = -2.28; BF_10_ > 10^21^), in architects (*t*_30_ = -15.11, *p* < .001, *d* = -2.71; BF_10_ > 10^12^), and in controls (*t*_25_ = -9.95, *p* < .001, *d* = -1.95; BF_10_ > 10^7^). Groups differed significantly (*t*_55_ = -2.08, *p* = .042, *d* = -0.55) with architects revealing larger distance effect, with anecdotal Bayesian support of the alternative hypothesis (BF_10_ = 1.59).

There is one outlier in architects (see Fig. S2). We repeated the analysis for the group without the outlier and found a robust distance effect on *z*-scored RTs (mean slope = -0.017, SD = 0.005; *t*_29_ = -17.62, *p* < .001, *d* = -3.22; BF_10_ > 10^14^) in this group, but the between-group difference was not significant any longer (*t*_54_ = -1.84, *p* = .071, *d* = -0.49; BF_10_ = 1.09).

*Fig S2. The distance effect for z-scored reaction time.*

Note also that our shared R scripts allow interested readers to calculate the remaining effects reported in the main manuscript and here using *z*-scored RTs. As the results are not substantially different from those obtained using raw RTs, we do not report them here.

**5. SNARC-like effect depending on the angle orientation**

In half of the trials of each task, the arms of the angles presented to participants were facing upwards, and in the other half, they were facing downwards. Although we did not explicitly pose any directional hypotheses about the orientation of the angles, we inspected the SNARC-like effect for upward and downward angles separately. The results are summarized in Table S2. In frequentist analyses, we did not find a significant SNARC-like effect for any angle orientation in any group in any task, and the Bayesian analyses substantially supported the null. In any case, we did not find significant between-group differences in the slopes in frequentist independent samples *t*-tests. The Bayesian analysis showed substantial evidence for the null except for upward-oriented angles in the indirect task where the effect was inconclusive. The direct task – upward orientation: *t*_55_ = -0.10, *p* = .921, *d* = -0.03; BF_01_ = 3.70. The direct task – downward orientation: *t*_55_ = 0.22, *p* = .826, *d* = 0.06; BF_01_ = 3.64. The indirect task – upward orientation: *t*_58_ = 1.70, *p* = .094, *d* = 0.44; BF_01_ = 1.14. The indirect task – downward orientation: *t*_58_ = 0.73, *p* = .467, *d* = 0.19; BF_01_ = 3.04.

**6.** **SNARC-like effect for the first half of trials of each task**

The SNARC-like effect observed by Fumarola et al. ^6^ could be a transitory phenomenon. According to this interpretation, participants could represent angles spatially in some initial trials and then switch to another representational format. While Fumarola et al. used 160 trials per task, in our procedure, we doubled this number (i.e., 320 trials per task). To make our results more directly comparable to those reported by Fumarola et al., we repeated analyses of SNARC-like effects for the first half of trials in each block of each task.

The results are summarized in Table S3. Frequentist analyses of data from both tasks did not reveal a significant SNARC-like effect at the whole sample level nor in each group separately. Bayesian analyses delivered substantial evidence for the null hypothesis in each case. In frequentist analyses, we did not find between-group differences in SNARC-like slopes in any case (direct task: *t*_54_ = 0.30, *p* = .767, *d* = 0.08; indirect task: *t*_57_ = 0.52, *p* = .602, *d* = 0.14). Bayesian analyses revealed substantial evidence for the nulls (direct task: BF_01_ = 3.55; indirect task: BF_01_ = 3.37). Note that degrees of freedom differ from those occurring in previously reported analyses since here we excluded different numbers of participants based on the same threshold of 70% valid trials (which equals 112 trials here).

We conclude that the failed replication of Fumarola et al.’s study is not due to the procedural difference, namely, doubling the number of trials.

*Table S3*. SNARC-like effect for the first half of trials of each task

| Group | Direct task | | | | Indirect task | | | |
| --- | --- | --- | --- | --- | --- | --- | --- | --- |
|  | Mean (SD) | *t*-test | *d* | BF_01_ | Mean (SD) | *t*-test | *d* | BF_01_ |
| whole sample | -0.01  (1.47) | *t*_55_ = -0.06,  *p* = .523 | -0.01 | 7.17 | -0.02 (0.37) | *t*_58_ = -0.42  *p* = .662 | -0.05 | 9.46 |
| architects | 0.04 (1.54) | *t*_30_ = 0.15,  *p* = .441 | 0.03 | 4.63 | <0.01 (0.38) | *t*_29_ = -0.07,  *p* = .473 | 0.01 | 4.88 |
| controls | -0.08 (1.41) | *t*_24_ = -0.27,  *p* - .606 | -0.05 | 5.76 | -0.04 (0.37) | *t*_28_ = -0.67  *p* = .747 | -0.13 | 7.87 |

*Notes.* *t*-test = one-sample *t*-test against zero (one-sided); *d* = Cohen’s *d*; BF = Bayes factor; BF_01_ values are provided (BF_01_ = 1/BF_10_).

The scripts for all the analyses are shared at the OSF (https://osf.io/ycdr9/)

**Referenes**

1. Halberda, J., Mazzocco, M. M. M. & Feigenson, L. Individual differences in non-verbal number acuity correlate with maths achievement. *Nature* **455**, 665–668 (2008).

2. Price, G. R., Palmer, D., Battista, C. & Ansari, D. Nonsymbolic numerical magnitude comparison: Reliability and validity of different task variants and outcome measures, and their relationship to arithmetic achievement in adults. *Acta Psychol. (Amst.)* **140**, 50–57 (2012).

3. Parkman, J. M. Temporal aspects of digit and letter inequality judgments. *J. Exp. Psychol.* **91**, 191–205 (1971).

4. Hohol, M. *et al.* Professional mathematicians do not differ from others in the symbolic numerical distance and size effects. *Sci. Rep.* **10**, (2020).

5. Fias, W., Lammertyn, J., Reynvoet, B., Dupont, P. & Orban, G. A. Parietal representation of symbolic and nonsymbolic magnitude. *J. Cogn. Neurosci.* **15**, 47–56 (2003).

6. Fumarola, A. *et al.* The spatial representation of angles. *Perception* **45**, 1320–1330 (2016).
